# Precise GNSS Positioning Using Smart Devices

**DOI:** 10.3390/s17102434

**Published:** 2017-10-24

**Authors:** Eugenio Realini, Stefano Caldera, Lisa Pertusini, Daniele Sampietro

**Affiliations:** Geomatics Research & Development s.r.l. (GReD), via Cavour 2, c/o ComoNExT, 22074 Lomazzo (Como), Italy; stefano.caldera@g-red.eu (S.C.); lisa.pertusini@g-red.eu (L.P.); daniele.sampietro@g-red.eu (D.S.)

**Keywords:** GNSS, smart devices, precise positioning

## Abstract

The recent access to GNSS (Global Navigation Satellite System) phase observations on smart devices, enabled by Google through its Android operating system, opens the possibility to apply precise positioning techniques using off-the-shelf, mass-market devices. The target of this work is to evaluate whether this is feasible, and which positioning accuracy can be achieved by relative positioning of the smart device with respect to a base station. Positioning of a Google/HTC Nexus 9 tablet was performed by means of batch least-squares adjustment of L1 phase double-differenced observations, using the open source goGPS software, over baselines ranging from approximately 10 m to 8 km, with respect to both physical (geodetic or low-cost) and virtual base stations. The same positioning procedure was applied also to a co-located u-blox low-cost receiver, to compare the performance between the receiver and antenna embedded in the Nexus 9 and a standard low-cost single-frequency receiver with external patch antenna. The results demonstrate that with a smart device providing raw GNSS phase observations, like the Nexus 9, it is possible to reach decimeter-level accuracy through rapid-static surveys, without phase ambiguity resolution. It is expected that sub-centimeter accuracy could be achieved, as demonstrated for the u-blox case, if integer phase ambiguities were correctly resolved.

## 1. Introduction

The use of single-frequency GNSS (Global Navigation Satellite System) receivers and antennas for high-precision static applications, such as geodetic monitoring, is becoming more and more important due to the extremely low cost of single-frequency hardware, originally designed and produced for the mass-market, and to the increasing number of GNSS systems available. Such applications require, in order to reach millimeter-level accuracy, the deployment of stationary GNSS units, delivering raw observations, and the processing of sufficiently short baselines [1,2,3,4,5,6,7]. However, for more traditional surveying operations, such as map updates [8], cadastral or archaeological surveys [9,10], or surveys to support other measurements, e.g., gravimetric networks [11], significantly lower accuracies, e.g., of the order of tens of centimeters, are sufficient. This accuracy level can be easily reached by single-frequency GNSS receiver observations, by means of acquisitions performed in a fast static mode (e.g., over a timespan of 10–15 min), opportunely elaborated in a classic relative positioning processing, i.e., by double-differencing observations with those coming from a GNSS CORS (Continuously Operating Reference Station). In this framework, the availability of GNSS receivers integrated in the so-called smart devices, such as smartphones or tablets, represents an important evolution. In fact, in principle, they can substitute complex and expensive instruments such as traditional GNSS receivers, total stations or theodolites in the above applications. In this sense, several studies were performed [12,13], but they were all limited in the positioning accuracy by the fact that no raw GNSS data were available in output, but only solutions internally computed by the GNSS module, thus not allowing for precise positioning.

Pesyna et al. [14] first demonstrated that centimeter-level precision could be achieved with a smartphone-quality GNSS antenna, the signal of which was redirected to an external receiver to generate range observations. These were then post-processed by code and phase double differences, achieving centimeter-level positioning. However, their results were limited to the demonstration of the practical usability of a smartphone-quality GNSS antenna: the signal acquisition and tracking, to generate code and phase observations, was still performed with an external radio frequency front-end and GNSS receiver, which overcame the limitations of the phone’s internal chipset and clock. Recently, some devices equipped with the latest Android operating system started to provide access to GNSS raw data directly from the embedded chipset, thus making it possible to apply advanced processing techniques and therefore improving the positioning solutions. In May 2016, during the “I/O 2016” conference, Google announced the possibility to retrieve GNSS raw data from smartphones and tablets equipped with Android Nougat (version 7) operating system or later [15]. At the end of 2016, the GNSS community started publishing first experiments of GNSS raw data logging from smart devices (see for instances the BlackDotGNSS blog [16] or the news reported in the Rokubun company website [17]). The main limiting factor in order to use smart devices with standard GNSS positioning software was due to the fact that the Google-developed logger does not allow to directly save raw data in RINEX (Receiver Independent Exchange)-compatible format but only to retrieve cumulated delta range expressed in terms of a constant (i.e., the wavelength) that multiplies the carrier phase (expressed in cycles) [17]. At the beginning of August 2017, the Geo++ company released a new app for Android systems, called Geo++ RINEX Logger, which allows one to log GPS, GLONASS and Galileo raw data acquired from smart devices and store it in RINEX format [18]. Another limiting factor for precise positioning with smart devices is represented by the so called duty cycling, i.e., a procedure used by GNSS modules embedded in smart devices to increase battery life. It basically consists in continuously alternating short periods (of the order of hundreds of milliseconds) in which the GNSS tracking is active, with periods in which it is disabled, thus continuously introducing discontinuities in phase observations. Among the first available Android-equipped devices allowing for GNSS raw data logging, the Nexus 9 tablet has duty cycling disabled.

In this work we present results of experiments performed to study the positioning accuracy achievable by means of the GNSS receiver and antenna integrated into a Nexus 9 tablet, aiming to prove the possibility to perform accurate rapid-static positioning with smart devices, without external components at the user’s location (the only external component of the system being a reference CORS). Note that results have been computed by means of the free and open source goGPS software [19,20], thus opening the possibility for a real low-cost solution to fast, decimeter-level surveys.

In the next section, the experiment design, a brief description of the GNSS data processing, results and experimental conclusions are outlined. The obtained results are discussed in Section 3.

## 2. Experiment

### 2.1. Hardware Setup

**Figure 1:**
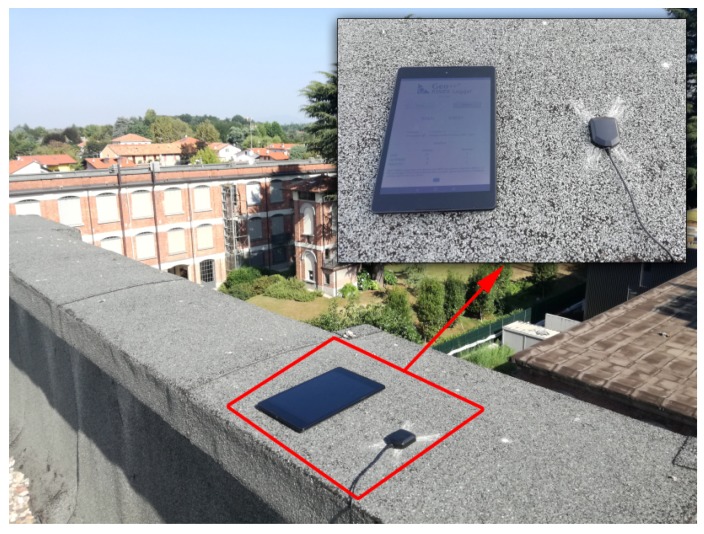
Nexus 9 tablet (**left**) and u-blox ANN-MS antenna (**right**) at the experiment location.

GNSS data were collected on 4 August 2017 over a timespan of 1.5 h by a Google/HTC Nexus 9 tablet (using the “Geo++ RINEX Logger” app), co-located with a u-blox EVK-6T receiver with its standard ANN-MS patch antenna (using the goGPS Java logger [21]—The RINEX files used in this work are freely available, upon request sent to info@g-red.eu.) The Nexus 9 tablet embeds a Broadcom BCM4752 GNSS receiver module, capable of tracking GPS, GLONASS and QZSS signals; in this work, GPS-only observations were used. The model and the location of the GNSS antenna within the Nexus 9 (size: 22 cm × 15 cm) is not known. Based on the information provided by the ifixit.com website (https://www.ifixit.com/Teardown/Nexus+9+Teardown/31425), it appears that an antenna board is located in the upper left corner of the device (when facing the display), but it is not clear whether that component includes the GNSS antenna as well. Both devices were located on the rooftop of a three-story building in Lomazzo (Como province, Italy), hosting also Geomatics Research & Development srl (GReD) headquarters (see Figure 1).

**Figure 2:**
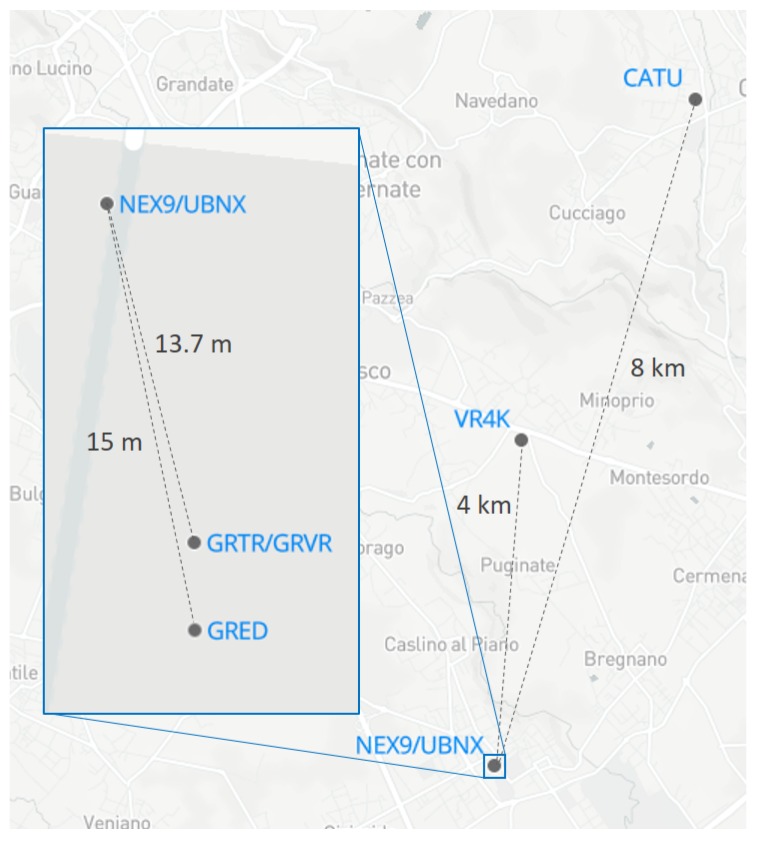
Stations used in the experiment (© Mapbox, Data ODbL © OpenStreetMap contributors).

On the same rooftop, at about 13 m distance from the experiment area, two CORSs are available, managed by GReD. These two stations include a multi-frequency, multi-constellation Trimble BD930 receiver connected to a Trimble Zephyr antenna, and a GeoGuard monitoring unit (GMU) [7], which features a single-frequency, multi-constellation u-blox LEA-M8T receiver connected to a Tallysman TW3470 antenna. The Trimble and GMU units were used as base stations for very short baseline experiments. A CORS belonging to the NetGeo (http://www.netgeo.it/) network, with marker name CATU, located in the town of Cantù, about 8 km distance from the experiment area, was also included to perform a short baseline test in a more realistic scenario. CATU station is equipped with a Topcon Net-G3 receiver and Topcon G3-A1 antenna. Finally, two Virtual Reference Stations (VRS) were generated by means of the SPIN positioning service (https://www.spingnss.it/): one at the same coordinates as the Trimble BD930 receiver (called GRVR), and one at 4 km distance from the experiment site (called VR4K), to check the positioning performance simulating the correction of positioning service network. All CORS locations are reported in Figure 2.

Summarizing, the coordinates of the following two devices were estimated (in brackets, the marker name chosen for the device):

- a Nexus 9 tablet (NEX9);
- a u-blox receiver (UBNX);

by relative positioning with respect to the following base stations (in order of baseline length; marker name in brackets):

- a Trimble receiver, 13.7 m baseline (GRTR);
- a SPIN VRS, 13.7 m baseline (GRVR);
- a GeoGuard GMU, 15 m baseline (GRED);
- a SPIN VRS, 4 km baseline (VR4K);
- the CATU NetGeo CORS, 8 km baseline (CATU).

### 2.2. GNSS Data Processing

The collected data were processed by the goGPS MATLAB software (https://github.com/goGPS-Project), that is an open source GNSS processing suite developed since 2007 by the Department of Civil and Environmental Engineering of Politecnico di Milano and by the authors of this paper [19,20]. Version 0.5.1 beta 3 of the goGPS MATLAB code was used. Caldera et al. [5] showed that goGPS solutions over short baselines, and using a low-cost receiver, reach the same level of accuracy and repeatability of the state-of-the-art Bernese GNSS Software. In the present work, a batch least-squares adjustment approach was chosen, to estimate the u-blox and Nexus 9 coordinates with respect to each of the five base stations. Double-differenced GPS-only L1 phase observations and broadcast ephemeris and satellite clocks were used for the solution adjustment. GPS-only L1 code pseudoranges and Doppler shifts were used in the pre-processing algorithms, to compute a-priori coordinates and to detect cycle-slips. A-priori coordinates for the u-blox and Nexus 9 devices were in fact estimated by stand-alone positioning using observed code pseudoranges: no a-priori information on the position of the devices was introduced into the processing. Cycle-slip detection was performed by a combination of Doppler-phase comparison and third-order phase derivative methods for the low-cost L1-only receivers (i.e., NEX9, UBNX, GRED); a combination of Geometry Free-based detection and Melbourne Wübbena-based detection was instead used for all dual-frequency datasets (i.e., GRTR, GRVR, VR4K, CATU). It should be pointed out that goGPS automatically chooses the most suited method to detect cycle-slips, based on input data. Ionospheric delays are computed by the Klobuchar model [22], using the broadcast parameters, and tropospheric delays by the Saastamoinen model [23], using standard atmosphere parameters, although with the short baselines used in this work they are both basically canceled by double-differences. The satellite elevation cut-off was set to 15 degrees, the observation weighting model was based on the sine of the elevation squared. The processing rate was set to match the available maximum observation rate for each baseline, i.e., 5 s for the GRTR and GRED stations, 1 s for the GRVR and VR4K virtual stations, and 30 s for the CATU CORS. As for the phase ambiguity resolution, goGPS uses the LAMBDA 3.0 MATLAB code developed by Verhagen et al. [24]; both float and LAMBDA-based fixed solutions were computed and evaluated. For each baseline connecting each of the five base stations to the u-blox and Nexus 9 devices, a processing of the complete dataset of 1.5 h was executed, as well as the processing of six 15-min sessions, each processed independently from the others.

### 2.3. Experimental Results and Conclusions

Since the aim of the present work is mainly to evaluate the accuracy level that can be reached by means of rapid-static surveys (of the order of tens of minutes) with smart devices, the 1.5-h solutions with the different base stations were computed to be used as reference to evaluate the 15-min session results for both the Nexus 9 and the u-blox receivers. The differences among the u-blox 1.5-h solutions with the five base stations are of the order of 0.5 cm for the horizontal coordinates and are smaller than 1.7 cm in the vertical direction, as it was expected [6]. The Nexus 9 references are slightly worse, but in any case smaller than 2 cm in the three directions (see Table 1).

In order to roughly evaluate the consistency of the reference Nexus 9 estimated position, its distance from the u-blox antenna, computed from the two GPS-derived positions, was compared to the one physically obtained with a measuring tape during the survey. It should be underlined here that this is just an approximate check, due to the not exactly known antenna position within the Nexus 9 device. Considering the Nexus 9 dimensions, the effective distance between the u-blox antenna and the unknown location of the Nexus 9 antenna could range from a minimum of 19 cm and a maximum of 32 cm. The GPS-estimated distance of 23.5 cm is, therefore, reasonable since it falls within the expected range.

**Figure 3:**
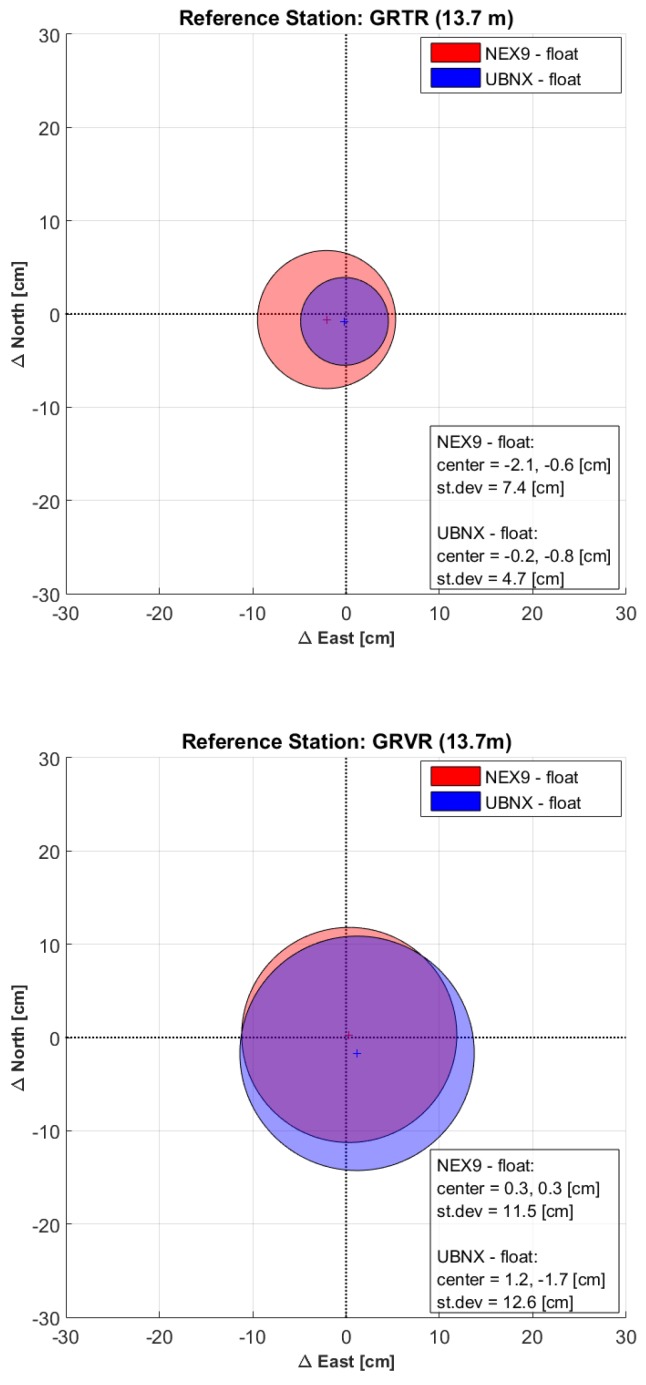

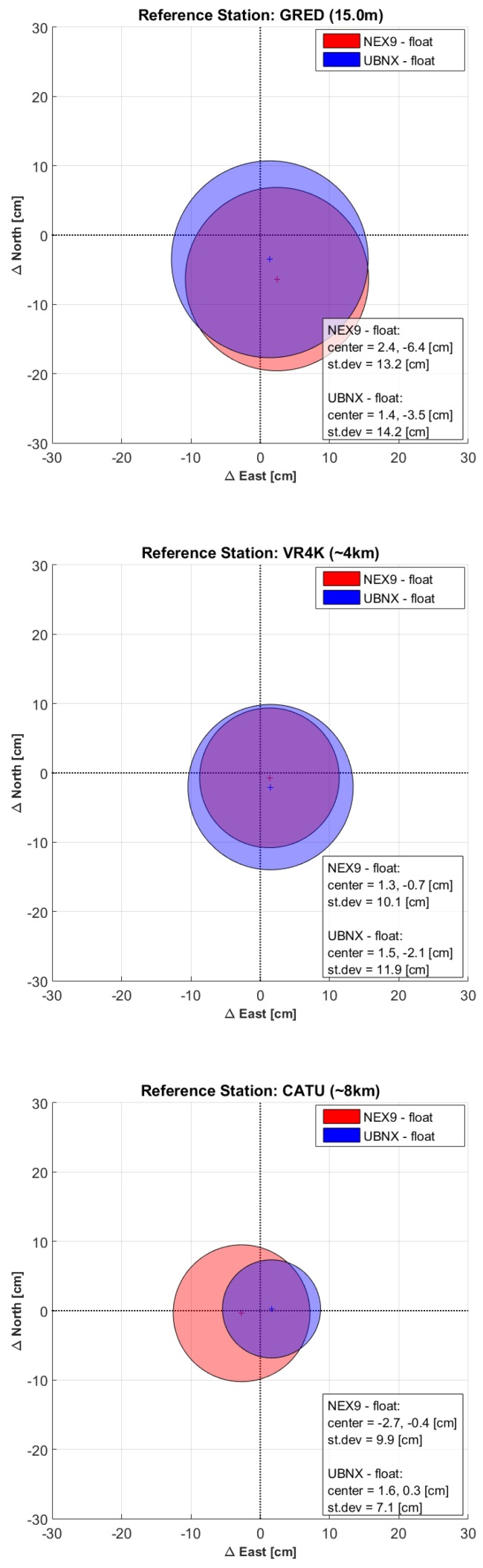
Bias (crosses) and 1-sigma dispersion (circles) of the differences between the 15-min float baselines of NEX9 and UBNX and their respective 1.5-h solution.

For each base station, the six 15-min solutions were compared to the corresponding 1.5-h one. When performing the above computations, problems arose in the Nexus 9 phase ambiguity resolution by the LAMBDA algorithm within the goGPS software: incorrect integer values were estimated, leading to larger errors for the fixed solutions compared to the float ones. On the contrary, phase ambiguities were always correctly solved for all the u-blox solutions. In Figure 3 the comparison between Nexus 9 and u-blox float solutions are shown.

It can be seen that the behaviors of the two receivers is similar; the u-blox performing slightly better when a geodetic physical receiver is used as base stations (GRTR and CATU), and the Nexus 9 when virtual stations or a single-frequency receiver are used. In any case, the difference shows the same order of magnitude for both receivers, considering each baseline case. Quite surprisingly, the result with the best average solution was obtained with the Nexus 9 when using the GRVR reference station, with an average difference of just 0.3 cm in both the East and North directions. The most precise solution is obtained with the GRTR base station, which is a physical dual-frequency station with a very short baseline of only 13.7 m (standard deviations of 7.4 cm and 4.7 cm for NEX9 and UBNX respectively), just marginally deteriorated when extending the length of the baseline to 8 km (standard deviations of 9.9 cm and 7.1 cm for CATU-NEX9 and CATU-UBNX baselines respectively). The solutions with respect to GRVR, and VR4K have almost the same precision with standard deviations ranging from 10.1 cm to 12.6 cm for both NEX9 and UBNX. As for the GRED reference station, which gives accuracies just slightly worse than the other reference stations, it should be reminded here that it is the only base station equipped with a single-frequency, low-cost receiver. Even in this case, the obtained accuracy of the NEX9 receiver is of the order of few centimeters (standard deviation of 13.2 cm and bias of 2.4 cm and −6.4 cm in the East and North directions respectively).

**Figure 4:**
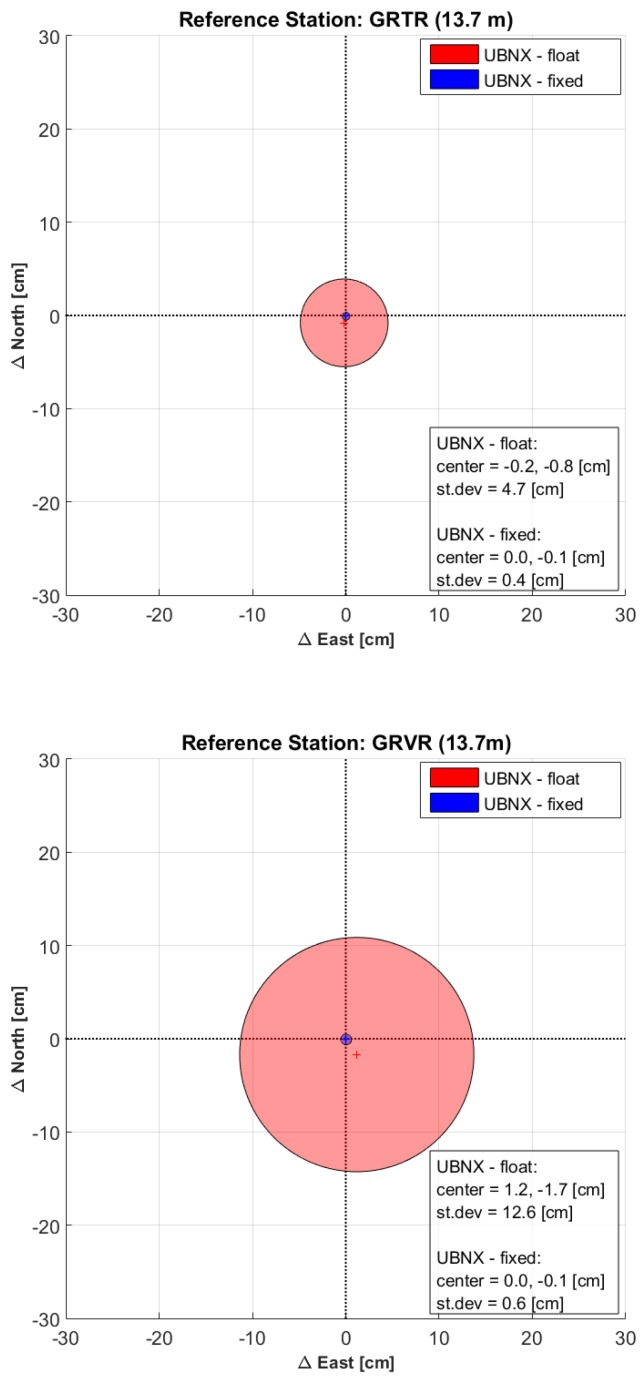

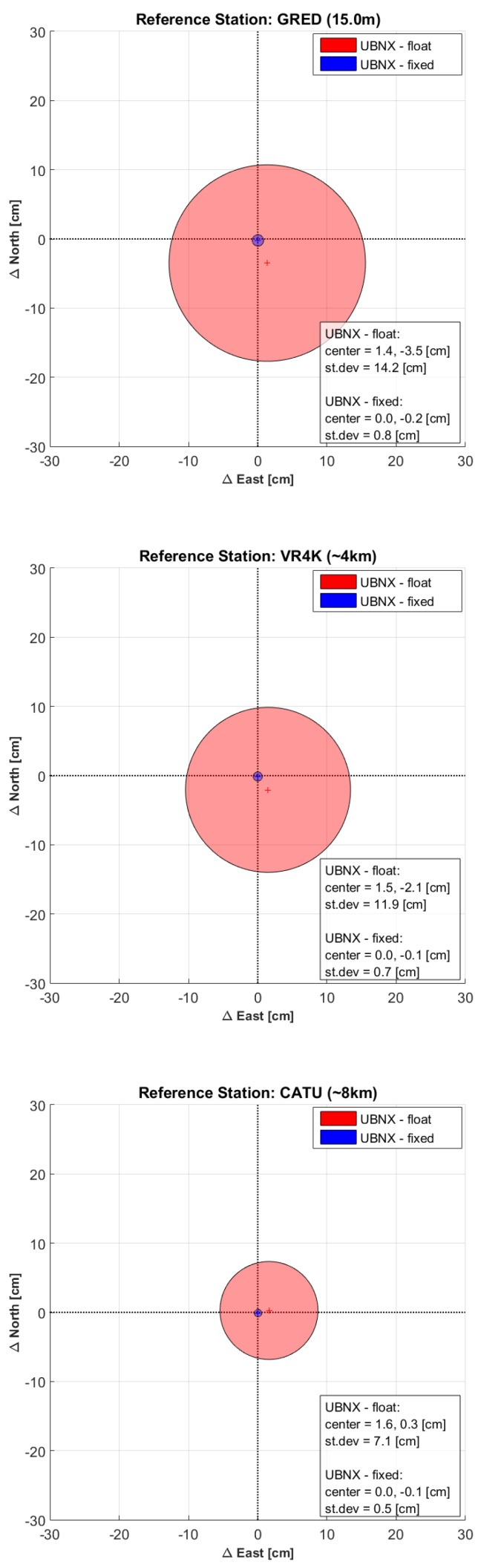
Bias (crosses) and 1-sigma dispersion (circles) of the differences between the 15-min float (red circles) and fixed (blue circles) baselines of UBNX and their respective 1.5-h solution.

In Figure 4, the improvements in the UBNX solution due to the integer phase ambiguity resolution is shown (compared to the UBNX float solution already shown if Figure 3). The bias drastically drops from few centimeters to about 0.1 cm. Similarly, the standard deviations improve of about one order of magnitude ranging between 0.4 cm and 0.8 cm for the ambiguity solved solution.

The above results are confirmed also for the vertical direction. The mean and standard deviation of the differences between the height values obtained by the 1.5-h solutions and the 15-min ones are reported in Table 2.

Again, the baselines between UBNX and the physical stations give the best results, with standard deviation of the order of 3.5 cm. The very short baseline GRTR-NEX9 also performs well with a standard deviation of 4.8 cm, while all the other baselines show a precision of the order of 10 cm. Also for the vertical direction, when the fixed solution is obtained (for the UBNX observations) both the bias and the standard deviation drastically improve to sub-centimeter level.

**Figure 5:**
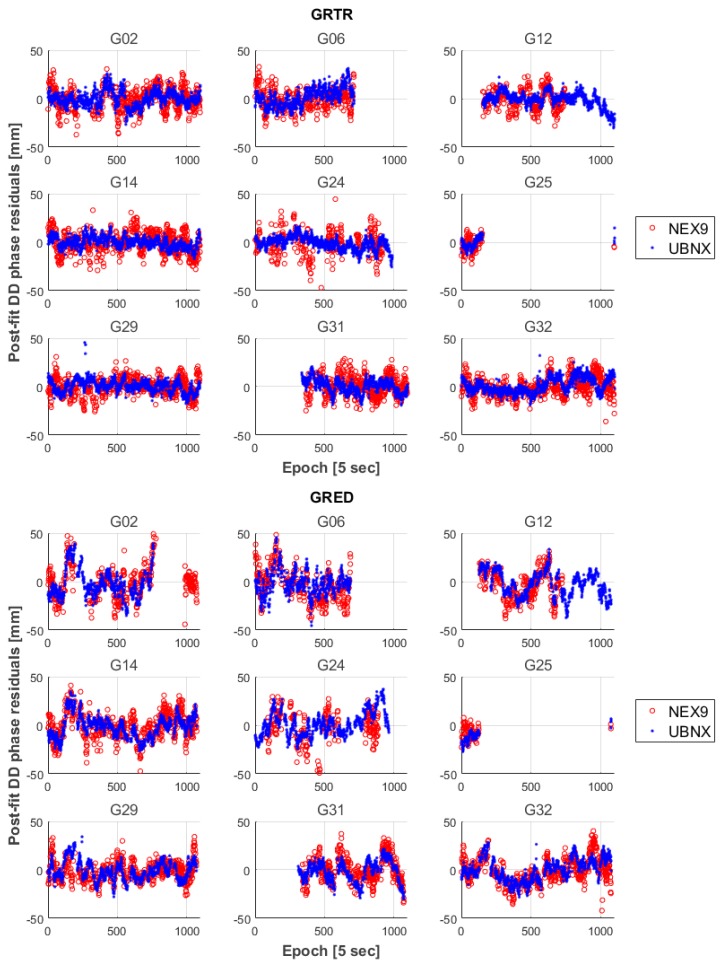
Post-fit double difference phase residuals of the float solutions for the baseline with respect to GRTR and GRED stations.

In Figure 5, the post-fit double-difference phase residuals for the baselines GRTR-NEX9, GRTR-UBNX, GRED-NEX9 and GRED-UBNX are shown. It can be seen that, for all baselines, the post-fit residuals of NEX9 are generally larger than those of UBNX in terms of dispersion, denoting a poorer performance of the NEX9 embedded antenna compared to the external UBNX one. However, both devices residuals show no significant bias. It can also be observed that residuals with respect to the GRTR and GRED base stations are quite similar for such a short baseline, notwithstanding the hardware quality (and cost) difference between the two base stations.

**Figure 6:**
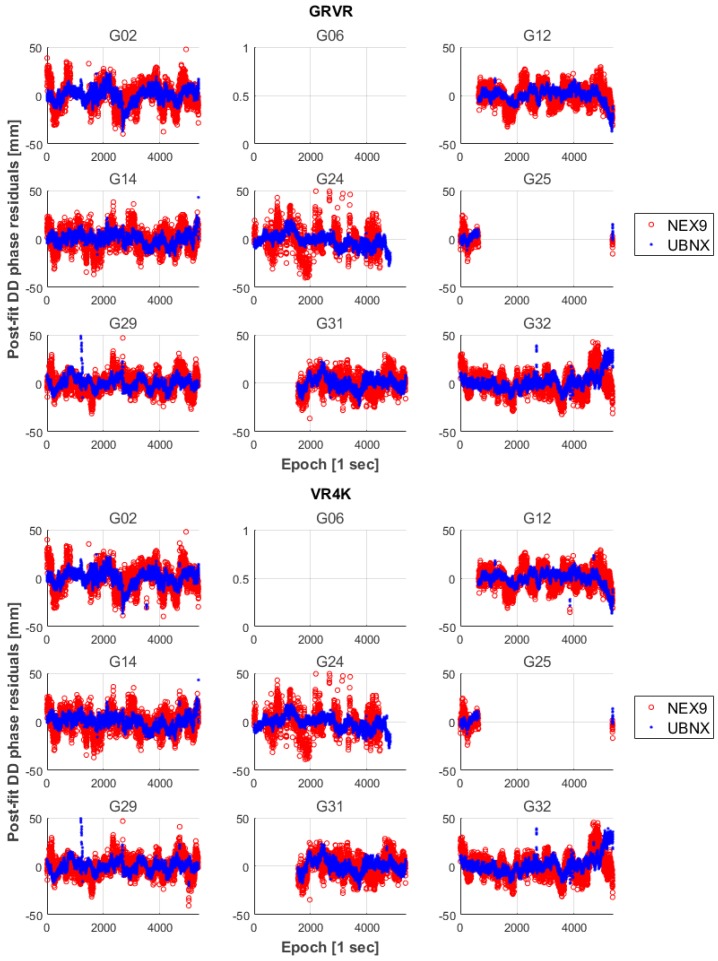
Post-fit double difference phase residuals of the float solutions for the baseline with respect to the two virtual stations.

Similarly, in Figure 6 the post-fit double difference phase residuals for the baselines with the two virtual stations (GRVR and VR4K) are shown.

It can be seen that the different lengths of the two baselines (13.7 m for the GRVR baseline and about 4 km for the VR4K one), do not have a significant effect on the phase residuals.

**Figure 7:**
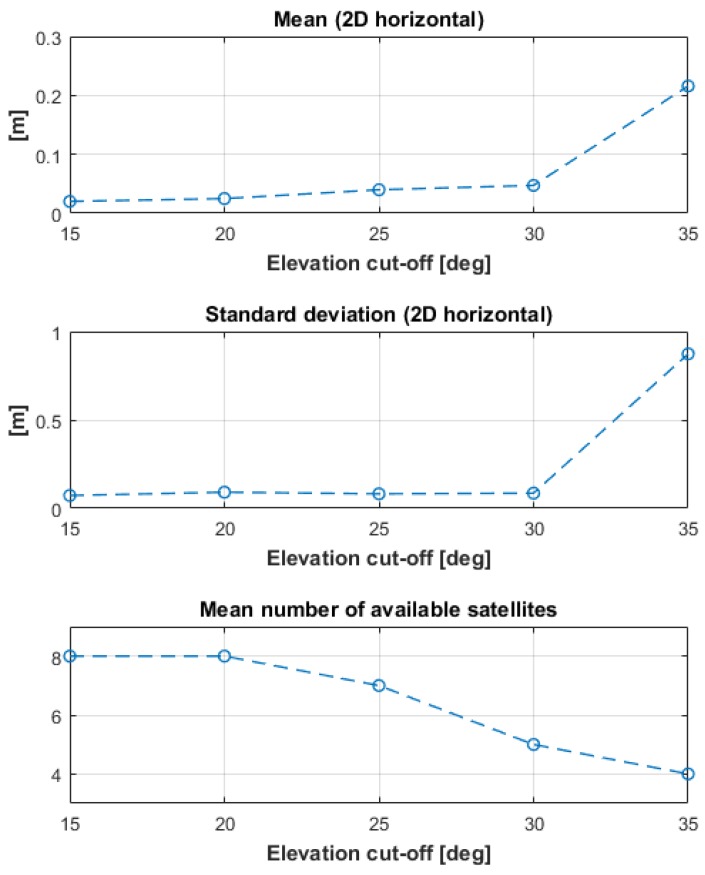
Solution degradation as the elevation cut-off angle is increased from 15 to 35 degrees.

To simulate a reduction of the number of visible satellites due to obstructions, the processing of the GRTR-NEX9 baseline float 15-min solutions was re-run, changing the elevation cut-off from 15 to 35 degrees, by 5 degree steps. The average number of satellites changes from eight for the 15 degree elevation cut-off, to four for the 35 degree elevation cut-off. The result is reported in Figure 7, showing that both mean and standard deviation of the differences from the 1.5 h float solution are below 10 cm up to 30 degrees of cut-off angle. The two indexes increase to about 20 cm (mean) and 1 m (standard deviation) when the cut-off angle reaches 35 degrees, and the average number of satellites goes down to four.

## 3. Discussion

As described in the previous section, carrier phase observations retrieved from a Nexus 9 device by means of the “Geo++ RINEX Logger” app show a quality similar to that of a standard single-frequency low-cost receiver. The performed experiments empirically demonstrated that smart devices, such as the Nexus 9, are suitable to be used to perform rapid-static surveys with decimeter-level accuracy. In this work, a precision of about 10 cm (standard deviation) on baselines up to about 8 km with 15 min float solutions was achieved. Even better results with sub-centimeter accuracy are expected in the future. In fact, the LAMBDA algorithm implemented in goGPS was not able to provide reliable integer phase ambiguities for the Nexus 9 observations. In principle, if one was able to correctly solve integer phase ambiguities, it would be possible to reach such a sub-centimeter accuracy, as demonstrated in the u-blox case. The Nexus 9 and u-blox float positioning solutions, as well as the post-fit phase residuals, are, in fact, basically consistent. Obviously, such an accuracy would be limited by the unknown exact location of the GNSS antenna within the smart device, but it is reasonable to expect that this issue will be solved.

As regards the solution dependence on the baseline length and on the base station type (i.e., geodetic versus low-cost, physical versus virtual), the performed experiment shows that, as expected, shorter baselines provide more accurate solutions, physical stations perform slightly better than virtual ones, and geodetic-grade bases better than low-cost ones. In general terms, all the tested solutions allow to achieve a positioning accuracy of the smart device better than 20 cm, and, in the case of physical geodetic stations, better than 10 cm. We conclude that the positioning capabilities of smart devices, when providing raw GNSS observations with continuous phase, are compatible with the requirements of decimeter-level accuracy survey applications.

The results achieved in this work, although preliminary (in that only float solutions were obtained with the smart device), open new perspectives in the effective use of such mass-market devices for precise positioning and professional surveying purposes. For the first time, it would be possible, in principle, to achieve centimeter-level accuracy with a completely low-cost solution, needing to have on the field just a smart device with its own embedded receiver and antenna.

## Figures and Tables

**Table 1:** Average coordinates obtained for the 1.5-h survey with different base stations on the first row (unit is meter, UTM (Universal Transverse Mercator) coordinate system), and differences between each solution and the average one (unit is centimeter).

Reference Station	UBNX			NEX9		
	East	North	Up	East	North	Up
Average Coord. [m]	502,747.189	5,060,416.944	308.819	502,746.993	5,060,417.073	308.809
GRTR [cm]	0.0	0.3	−1.7	−0.5	0.2	0.1
GRVR [cm]	−0.1	0.4	0.9	1.7	−0.3	2.0
GRED [cm]	0.3	−0.4	0.5	−2.1	0.7	−2.3
VR4K [cm]	−0.2	0.2	1.3	1.3	−0.2	1.5
CATU [cm]	0.0	−0.6	−1.0	−0.4	−0.5	−1.3

**Table 2:** Mean and standard deviation (std) of the differences in the vertical direction between the 15-min float baselines of NEX9 and UBNX and their respective 1.5-h solution.

Reference Station	UBNX		NEX9		UBNX Fixed	
	Mean	Std	Mean	Std	Mean	Std
GRTR [cm]	−0.1	3.4	−0.6	4.8	−0.1	0.3
GRVR [cm]	−0.2	9.4	0.3	8.8	−0.1	0.4
GRED [cm]	−3.5	10.1	−6.4	9.2	−0.2	0.6
VR4K [cm]	−2.1	9.0	−0.7	8.5	−0.1	0.3
CATU [cm]	0.3	3.5	−0.4	8.0	−0.1	0.2
